# Postoperative inflammatory markers and surgical wound infection after elective short-segment lumbar fusion: the importance of surgical context

**DOI:** 10.5194/jbji-11-503-2026

**Published:** 2026-08-18

**Authors:** Jay Chung, Ta'ir Rocker, Regina Golding, Sachin Mehta, Madison McFarland, Arjun Gupta, Xiuyi Yang, Priya Singh, Evan Olsen, Shreya Balusu, Yungtai Lo, Pamela Zuckerman, Jeremy D. Shaw, Mitchell S. Fourman

**Affiliations:** 1 Albert Einstein College of Medicine, Bronx, NY 10461, USA; 2 Department of Orthopedic Surgery, Montefiore Medical Center, Bronx, NY 10461, USA; 3 Intermountain Neurosciences, Intermountain Health, Salt Lake City, UT 84107, USA

## Abstract

**Introduction**: Postoperative white blood cell (WBC) count and neutrophil-to-lymphocyte ratio (NLR) are routinely available after lumbar fusion, but early elevations may reflect surgical stress. We evaluated associations of postoperative day (POD) 1 and POD3 WBC/NLR with 90 d surgical wound infection (SWI) after elective short-segment lumbar fusion. **Methods**: This retrospective cohort included adults undergoing elective one- to three-level lumbar fusion from 2022 to 2024 at one academic center. SWI was a pragmatic electronic-health-record-based endpoint encompassing clinically documented wound-related events, not a uniformly culture-confirmed deep, implant-associated, or surveillance-defined infection. Complete-case univariable and parsimoniously adjusted logistic regressions examined WBC/NLR associations with SWI. **Results**: Among 380 outcome-eligible patients, 18 (4.7 %) developed SWI. POD1 analyses included 368 patients (18 events), POD3 analyses 336 (17 events), and paired analyses 327 (17 events). After adjustment for body mass index and operative duration, POD1 associations were attenuated; POD3 WBC (odds ratio 1.17, 95 % confidence interval 1.00–1.37; p=0.049 ) and NLR (odds ratio 1.12, 95 % confidence interval 1.01–1.23; p=0.026 ) remained associated with SWI. However, these associations weakened after adjustment for corresponding POD1 values. Absolute and percentage POD1-to-POD3 changes were not significantly associated with SWI. **Conclusions**: POD1 WBC/NLR elevations were associated with patient and operative context. POD3 values showed exploratory associations with SWI after limited adjustment but did not establish diagnostic thresholds, clinical utility, or a validated prediction model.

## 1 Introduction

Surgical wound infection (SWI) after lumbar spine surgery is a potentially catastrophic complication, with reported incidence varying by patient and procedural factors (Zhou et al., 2020). SWI is associated with prolonged hospitalization, perioperative morbidity, 90 d readmission, revision surgery, pain, disability, and financial burden (Suszczynska et al., 2025; Elsamadicy et al., 2024). Clarifying how routine inflammatory markers relate to SWI may improve interpretation of postoperative laboratory abnormalities.

Routine laboratory markers such as white blood cell (WBC) count and neutrophil-to-lymphocyte ratio (NLR) have been associated with postoperative infection in prior studies (Shen et al., 2019; Qian et al., 2023; Iwata et al., 2016). However, early WBC and NLR elevations may reflect operative stress, patient physiology, comorbidity, body mass index (BMI), extent of tissue dissection, number of operative levels, and operative duration rather than infection-specific biology (Watt et al., 2015; Kobayashi et al., 2020; Liu et al., 2018; Schoenberg et al., 2023).

In this study, SWI was defined as a pragmatic electronic-health-record-based endpoint intended to capture clinically documented wound-related events. It was not equivalent to a uniformly culture-confirmed deep infection, implant-associated infection, or standard surveillance-defined surgical site infection (SSI).

This study evaluated whether POD1 and POD3 WBC/NLR remained associated with SWI after accounting for patient and operative factors that influence postoperative inflammation.

## 2 Methods

A retrospective review was performed of prospectively collected data from adult patients who underwent elective one- to three-level lumbar fusion from 2022 to 2024 at a single academic medical center. A source registry of 462 cases was reviewed. After data cleaning and construction of the completed WBC/NLR abstraction dataset, 380 patients comprised the outcome-eligible analytic cohort. Data were obtained from the institutional electronic health record, including operative reports, laboratory records, postoperative encounters, emergency department visits, readmissions, and reoperation documentation.

Patients were included if they were 18 years of age or older, underwent elective one- to three-level lumbar fusion from 2022 to 2024, had 90 d outcome ascertainment, and had postoperative complete blood count (CBC) data available for at least one relevant inflammatory-marker analysis. Patients were excluded if follow-up was insufficient to ascertain the primary outcome, if surgery was performed for oncologic or infectious indications, or if surgery was emergent. Laboratory analyses used complete-case availability for each time point: POD1 analyses included patients with complete POD1 WBC, absolute neutrophil count (ANC), absolute lymphocyte count (ALC), and NLR values; POD3 analyses included patients with complete POD3 values; and paired trajectory analyses included only patients with complete POD1 and POD3 values.

Baseline demographic and clinical variables included age, sex, BMI, obesity class, diabetes, smoking status, race/ethnicity, insurance category, age-adjusted Charlson Comorbidity Index, and Area Deprivation Index (Petterson, 2023). Operative variables included approach, number of fused levels, operative duration, estimated blood loss, transfusion, prior laminectomy, adjacent-level laminectomy, and intraoperative complication. Postoperative variables included length of stay, ICU/step-down admission, discharge disposition, 90 d emergency department visit, readmission, and return to the operating room.

The primary laboratory values of interest were postoperative inflammatory markers measured as WBC and NLR on POD1 and POD3. Laboratory values were collected per standard hospital protocol. NLR was calculated as ANC divided by ALC from the corresponding CBC differential. When more than one laboratory value was available for a given postoperative day, the value closest to the target time point (24 h for POD1 and 72 h for POD3) was used.

The primary outcome was SWI within 90 d of surgery, defined pragmatically from the electronic health record as a documented clinical wound infection or wound-related infectious complication requiring antibiotic therapy, wound intervention, and/or reoperation. Infection status was confirmed through postoperative inpatient and outpatient encounters, emergency department visits, readmissions, wound evaluations, and operative documentation when applicable. This pragmatic endpoint was not equivalent to a uniformly culture-confirmed deep infection, implant-associated infection, or standard CDC/NHSN- or ACS-NSQIP-defined SSI. SWI cases were further categorized by treatment phenotype as return to OR, nonoperative wound complication/intervention, or nonoperative/antibiotic-only clinical treatment.

All statistics were performed using SPSS 30.0.0 (IBM Corp., Armonk, NY, USA). Continuous variables were compared using Mann–Whitney U tests, and categorical variables were compared using chi-square or Fisher exact tests, as appropriate. Within-patient POD1-to-POD3 changes were evaluated using paired t tests. Logistic regression estimated odds ratios (ORs) with 95 % confidence intervals (CIs), and statistical significance was defined as two-sided p<0.05 .

To evaluate potential selection bias related to POD3 laboratory availability, patients with complete paired POD1–POD3 WBC/ANC/ALC/NLR values were compared with outcome-eligible patients who lacked complete paired laboratory data.

Trajectory analyses were performed among patients with complete paired POD1 and POD3 WBC/ANC/ALC/NLR values. Absolute change was calculated as POD3 minus POD1, and percentage change was calculated as absolute change divided by POD1. POD1 values, POD3 values, absolute changes, and percentage changes were compared by SWI status.

Because only 18 SWI events occurred, multivariable logistic regression models were kept parsimonious to reduce overfitting. Models focused on prespecified covariates, including BMI and operative duration, while additional covariates were evaluated descriptively. A sensitivity analysis restricted to the more definite return-to-OR/washout phenotype was considered. Because only nine such events occurred, regression modeling for this restricted phenotype was not performed because estimates would be unstable and overfit.

## 3 Results

### 3.1 Cohort selection and laboratory missingness

Of the total 462 screened source-registry cases, 380 patients were included in the outcome-eligible WBC/NLR analytic cohort. Eighteen patients developed 90 d SWI and 362 did not. Laboratory analyses used complete-case denominators specific to the marker and time point. POD1 WBC/ANC/ALC/NLR values were complete in 368 patients (18 SWI events), POD3 values were complete in 336 patients (17 SWI events), and paired POD1–POD3 values were complete in 327 patients (17 SWI events) (Table 1).

**Table 1:** Cohort flow and laboratory analysis denominators.

Cohort step	Patients, n	Notes
Source registry reviewed	462	Elective one- to three-level lumbar fusion cases from 2022 to 2024 in the source registry
Outcome-eligible analytic cohort	380	Patients carried forward into the completed WBC/NLR abstraction dataset with 90 d outcome ascertainment
90 d SWI	18	4.7 % of outcome-eligible cohort
No 90 d SWI	362	95.3 % of outcome-eligible cohort
Complete POD1 WBC/ANC/ALC/NLR	368	POD1 complete-case denominator: 18 SWI and 350 no SWI
Complete POD3 WBC/ANC/ALC/NLR	336	POD3 complete-case denominator: 17 SWI and 319 no SWI
Complete paired POD1–POD3 values	327	Paired trajectory and sensitivity denominator: 17 SWI and 310 no SWI

### 3.2 Baseline and operative characteristics

Patients who developed SWI had higher BMI, more frequent morbid obesity, and longer operative duration. Other demographic, laboratory, and operative characteristics were not significantly different by SWI status (Table 2).

**Table 2:** Baseline demographics and operative characteristics by SWI status.

Characteristic	No SWI ( n=362 )	SWI ( n=18 )	P value
Age, years	62.6 (55.6–69.1)	58.0 (52.0–63.1)	0.104
Female sex	256/362 (70.7 %)	13/18 (72.2 %)	1.000
BMI, kg m^−2^	31.0 (27.3–34.8)	35.3 (33.1–39.9)	< 0.001
Morbid obesity	88/362 (24.3 %)	9/18 (50.0 %)	0.024
Active smoker	57/362 (15.7 %)	4/18 (22.2 %)	0.507
Diabetes	115/362 (31.8 %)	6/18 (33.3 %)	1.000
National ADI	27.0 (19.0–33.0)	27.0 (21.0–29.0)	0.913
NY ADI	6.0 (5.0–7.0)	6.0 (5.0–6.0)	0.915
Age-adjusted CCI	3.0 (2.0–4.0)	3.0 (2.0–4.0)	0.408
Preoperative WBC, $\times 10^{9}$ L^−1^	6.6 (5.3–8.1)	6.9 (5.2–8.1)	0.755
Preoperative NLR	1.7 (1.2–2.5)	1.8 (1.2–2.5)	0.998
Prior laminectomy	65/362 (18.0 %)	4/18 (22.2 %)	0.753
Adjacent-level laminectomy	18/362 (5.0 %)	1/18 (5.6 %)	0.611
Any intraoperative complication	24/362 (6.6 %)	3/18 (16.7 %)	0.127
Intraoperative transfusion	5/362 (1.4 %)	1/18 (5.6 %)	0.254
Estimated blood loss, mL	200.0 (100.0–250.0)	200.0 (56.2–300.0)	0.438
Operative time, min	263.5 (207.0–351.0)	368.0 (312.0–449.5)	< 0.001
ICU/step-down postoperative admission	19/362 (5.2 %)	0/18 (0.0 %)	1.000
Hospital LOS, days	4.0 (3.0–6.0)	4.0 (3.0–5.0)	0.629
Discharge to SNF/rehab	71/362 (19.6 %)	1/18 (5.6 %)	0.216
Surgical approach, overall			0.838
Open	204/362 (56.4 %)	10/18 (55.6 %)	
MIS posterior	82/362 (22.7 %)	5/18 (27.8 %)	
Anterior/ALIF/OLIF	76/362 (21.0 %)	3/18 (16.7 %)	
Lumbar levels, overall			0.157
1 level	202/362 (55.8 %)	6/18 (33.3 %)	
2 levels	115/362 (31.8 %)	8/18 (44.4 %)	
3 levels	45/362 (12.4 %)	4/18 (22.2 %)	

Values are median (interquartile range) for continuous variables and n/N (%) for categorical variables. P values are from Mann–Whitney U tests for continuous variables and Fisher exact or chi-square tests for categorical variables as appropriate.

### 3.3 Clinical characterization of SWI events

Among the 18 patients classified as having 90 d SWI, nine underwent return to the operating room for wound exploration, washout, irrigation and debridement, or related operative wound management. Seven additional patients had nonoperative wound-related complications or interventions documented in emergency department or readmission records, including incisional drainage, wound dehiscence, wound-vacuum/prevena management, abscess, operative-site bleeding, or wound pain/discharge. Two patients were classified as nonoperative/antibiotic-only or clinically treated SWI cases because no return to OR or procedural wound intervention was documented in the available dataset (Table 3). Because only nine return-to-OR/washout events occurred, no regression sensitivity analysis was performed for this restricted phenotype.

**Table 3:** Clinical characterization of 90 d SWI events by treatment phenotype.

Case	Documented wound-related evidence in dataset	Treatment phenotype	Relevant utilization/intervention
1	SWI marked; no ER/readmission/return-OR reason documented	Nonoperative/antibiotic-only or clinically treated SWI	No documented wound procedure in dataset
2	ER visit for pain and oozing from surgical site	Nonoperative wound complication/intervention	ED wound evaluation
3	Return to OR for washout; MSSA bacteremia postop	Return to OR/washout	Operative washout; MSSA bacteremia noted
4	Readmission for drainage; wound vac/prevena issue; prevena placed	Nonoperative wound complication/intervention	Readmission/wound-vac management
5	Seroma, wound dehiscence, drainage/closure in ED	Nonoperative wound complication/intervention	ED closure/drainage management
6	Return to OR for non-healing wound/wound dehiscence	Return to OR/wound exploration	Operative wound exploration
7	Return to OR for spinal wound exploration, washout, wound vac	Return to OR/washout	Operative washout/wound-vac management
8	Return to OR for wound exploration/wound drainage	Return to OR/wound exploration	Operative wound exploration
9	Return to OR for wound exploration/washout for superficial dehiscence	Return to OR/washout	Operative washout
10	Readmission/ER for buttock abscess	Nonoperative wound complication/intervention	Abscess evaluation/treatment
11	ER for drainage from incision on POD7	Nonoperative wound complication/intervention	ED wound evaluation
12	ER for bleeding from operative site	Nonoperative wound complication/intervention	ED wound evaluation
13	Return to OR for spinal wound exploration/washout	Return to OR/washout	Operative washout
14	ER for wound pain/discharge and self-resolving fever	Nonoperative wound complication/intervention	ED wound evaluation
15	Return to OR for spinal wound exploration/washout; infection	Return to OR/washout	Operative washout
16	Return to OR for spine I&D, wound exploration, dura repair	Return to OR/I&D	Operative I&D/wound exploration
17	ER for back pain/postoperative problem; no wound procedure documented	Nonoperative/antibiotic-only or clinically treated SWI	No documented wound procedure in dataset
18	Return to OR for wound exploration; drainage from incision site	Return to OR/wound exploration	Operative wound exploration

Categories were assigned using the 90 d SWI flag, return-to-OR field, readmission/ER fields, and wound-related reason text. The intermediate category is labeled nonoperative wound complication/intervention because some cases had wound-related clinical documentation without a clearly documented procedure.

### 3.4 Selection bias from paired POD1–POD3 laboratory availability

Patients with complete paired POD1–POD3 WBC/ANC/ALC/NLR values ( n=327 ) were compared with outcome-eligible patients without complete paired laboratory data ( n=53 ). Paired-lab patients had longer operative duration, greater estimated blood loss, longer hospital stay, and more open procedures, while SWI, return to OR, readmission, and ED visit rates did not differ significantly.

**Table 4:** Comparison of patients with and without complete paired POD1–POD3 laboratory values.

Characteristic	Paired POD1–POD3 labs ( n=327 )	No complete paired labs ( n=53 )	P value
Age, years	62.4 (55.4–69.2)	61.5 (56.0–67.3)	0.699
Female sex	234/327 (71.6 %)	35/53 (66.0 %)	0.419
BMI, kg m^−2^	31.4 (27.4–35.3)	29.8 (27.0–32.8)	0.101
Operative time, min	279.0 (216.0–366.5)	225.0 (173.0–290.0)	< 0.001
Estimated blood loss, mL	200.0 (100.0–300.0)	100.0 (40.0–200.0)	< 0.001
Hospital LOS, days	4.0 (3.0–6.0)	2.0 (2.0–3.0)	< 0.001
SWI	17/327 (5.2 %)	1/53 (1.9 %)	0.488
Return to OR	11/327 (3.4 %)	1/53 (1.9 %)	1.000
Readmission	32/327 (9.8 %)	3/53 (5.7 %)	0.447
ED visit	59/327 (18.0 %)	8/53 (15.1 %)	0.700
Surgical approach, overall			< 0.001
Open	197/327 (60.2 %)	17/53 (32.1 %)	
MIS posterior	69/327 (21.1 %)	18/53 (34.0 %)	
Anterior/ALIF/OLIF	61/327 (18.7 %)	18/53 (34.0 %)	
Lumbar levels, overall			0.096
1 level	177/327 (54.1 %)	31/53 (58.5 %)	
2 levels	103/327 (31.5 %)	20/53 (37.7 %)	
3 levels	47/327 (14.4 %)	2/53 (3.8 %)	

Values are median (interquartile range) for continuous variables and n/N (%) for categorical variables. P values are from Mann–Whitney U tests for continuous variables and Fisher exact or chi-square tests for categorical variables as appropriate.

### 3.5 WBC/NLR values and trajectories

Among 327 patients with complete paired POD1 and POD3 values, 17 developed 90 d SWI and 310 did not. WBC and NLR decreased significantly from POD1 to POD3 across the paired cohort (WBC decline $1.57\times 10^{9}$ L^−1^, 95 % CI 1.28–1.86; NLR decline 4.54, 95 % CI 3.94–5.15; both p<0.001 ).

By SWI status, POD1 WBC, POD3 WBC, POD1 NLR, absolute WBC change, percentage WBC change, absolute NLR change, and percentage NLR change did not differ significantly. POD3 NLR was higher among patients who developed SWI (5.1 [4.3–8.9] vs. 3.7 [2.7–5.3], p=0.007 ).

**Table 5:** POD1, POD3, and trajectory inflammatory markers by SWI status among patients with paired laboratory values ( N=327 ; 17 events).

Marker	No SWI ( n=310 )	SWI ( n=17 )	P value
POD1 WBC, $\times 10^{9}$ L^−1^	11.6 (9.5–13.6)	12.7 (10.6–16.3)	0.157
POD3 WBC, $\times 10^{9}$ L^−1^	10.0 (8.0–11.6)	11.4 (8.7–16.4)	0.123
Delta WBC, POD3–POD1	- 1.5 ( - 3.2 to 0.0)	- 1.0 ( - 3.1 to 0.5)	0.621
% change WBC	- 13.4 ( - 26.9 to 0.0)	- 14.7 ( - 18.0 to 4.3)	0.541
POD1 NLR	7.6 (5.2–11.1)	9.5 (5.1–17.7)	0.327
POD3 NLR	3.7 (2.7–5.3)	5.1 (4.3–8.9)	0.007
Delta NLR, POD3–POD1	- 3.4 ( - 7.1 to - 1.1)	- 2.9 ( - 8.1 to - 0.4)	0.857
% change NLR	- 48.8 ( - 68.4 to - 24.6)	- 33.8 ( - 58.9 to - 9.9)	0.284

Values are median (interquartile range). Delta values are calculated as POD3–POD1; negative values indicate decline from POD1 to POD3. The complete-case denominator was N=327 , including 17 SWI events and 310 no-SWI patients.

### 3.6 Regression models

POD1 WBC, POD1 NLR, POD3 WBC, and POD3 NLR were associated with SWI in univariable models. After adjustment for BMI and operative time, POD1 associations were attenuated, whereas POD3 WBC and POD3 NLR remained associated with SWI. Sensitivity models adjusting POD3 values for corresponding POD1 values attenuated these associations; delta WBC and delta NLR were not significantly associated with SWI.

**Table 6:** Univariable, parsimoniously adjusted, and trajectory-sensitivity logistic regression models for 90 d SWI.

Predictor	Model	OR (95 % CI)	P value	Notes/covariates
POD1 WBC (per 1 $\times 10^{9}$ L^−1^)	Univariable	1.15 (1.00–1.31)	0.047	N=368 ; 18 events
POD1 WBC (per 1 $\times 10^{9}$ L^−1^)	Adjusted	1.10 (0.96–1.26)	0.180	BMI + operative time; N=368 ; 18 events
POD1 NLR (per unit)	Univariable	1.08 (1.01–1.14)	0.019	N=368 ; 18 events
POD1 NLR (per unit)	Adjusted	1.05 (0.98–1.12)	0.183	BMI + operative time; N=368 ; 18 events
POD3 WBC (per 1 $\times 10^{9}$ L^−1^)	Univariable	1.18 (1.03–1.36)	0.021	N=336 ; 17 events
POD3 WBC (per 1 $\times 10^{9}$ L^−1^)	Adjusted	1.17 (1.00–1.37)	0.049	BMI + operative time; N=336 ; 17 events
POD3 NLR (per unit)	Univariable	1.10 (1.00–1.22)	0.045	N=336 ; 17 events
POD3 NLR (per unit)	Adjusted	1.12 (1.01–1.23)	0.026	BMI + operative time; N=336 ; 17 events
POD3 WBC adjusted for POD1 WBC	Sensitivity	1.15 (0.94–1.41)	0.180	POD1 WBC + BMI + operative time; N=327 ; 17 events
POD3 NLR adjusted for POD1 NLR	Sensitivity	1.10 (0.99–1.22)	0.089	POD1 NLR + BMI + operative time; N=327 ; 17 events
Delta WBC (POD3-POD1)	Sensitivity	1.03 (0.85–1.23)	0.790	BMI + operative time; N=327 ; 17 events
Delta NLR (POD3-POD1)	Sensitivity	0.97 (0.91–1.05)	0.490	BMI + operative time; N=327 ; 17 events

Adjusted models included the marker of interest, BMI, and operative time unless otherwise noted. Odds ratios are exploratory association estimates and should not be interpreted as validated diagnostic thresholds. POD1 and POD3 markers were analyzed in separate models.

## 4 Discussion

In this retrospective cohort of adults undergoing elective short-segment lumbar fusion, POD3 WBC and NLR showed exploratory associations with 90 d SWI after parsimonious adjustment for BMI and operative time, whereas POD1 associations were attenuated after adjustment. These findings suggest that immediate postoperative inflammatory markers are strongly influenced by patient and operative context, while POD3 values may retain a residual exploratory association with SWI. The first 24 h after lumbar fusion are marked by leukocytosis and stress-associated differential shifts from tissue trauma, perioperative medications, hemodilution, physiologic stress, and obesity (Watt et al., 2015; Kobayashi et al., 2020; Liu et al., 2018). Isolated POD1 WBC or NLR elevation may therefore reflect surgical trauma rather than early infection.

NLR and WBC values typically begin to stabilize by postoperative day three to four as the acute inflammatory response resolves (Aono et al., 2025; Deirmengian et al., 2011). Prior work has reported associations between POD4 NLR/WBC and SWI in a heterogeneous lumbar surgery population (Shen et al., 2019). In the present cohort, POD3 elevation, particularly NLR, showed a stronger exploratory association with SWI than POD1 elevation, although interpretation still requires clinical context.

The trajectory analyses refined this interpretation. Although WBC and NLR declined significantly from POD1 to POD3, the magnitude of absolute or percentage decline did not differ by SWI status. In addition, the POD3 associations were attenuated when the corresponding POD1 value was included in sensitivity models. Thus, the most defensible trajectory-related observation was higher POD3 NLR among SWI patients, not failure of WBC/NLR to decline or independent clinical utility of the POD3 values.

These results should be interpreted cautiously. This study did not develop diagnostic thresholds, receiver operating characteristic analysis, calibration assessment, sensitivity, specificity, predictive values, or decision-curve analysis. The findings should not guide clinical protocols without prospective validation.

Postoperative spine infection definitions remain heterogeneous. CDC/NHSN and ACS-NSQIP definitions provide standardized surveillance categories but may not fully capture the clinical overlap between superficial wound problems, sterile dehiscence, deep infection, implant-associated infection, delayed presentation, and culture-negative infection after instrumented fusion (Alavi et al., 2025; Shaw et al., 2025; Warner et al., 2026). Recent postoperative spine infection frameworks emphasize integrating wound findings, microbiology, imaging, inflammatory markers, intraoperative findings, and histology when available (Alavi et al., 2025; Shaw et al., 2025). The present SWI definition was chosen to reflect real-world wound events captured in the electronic health record, but it should not be interpreted as a uniformly culture-confirmed deep infection, implant-associated infection, or standard surveillance-defined SSI. For transparency, events were further categorized as return to OR, nonoperative wound complication/intervention, or nonoperative/antibiotic-only clinical treatment.

Prior spine studies have often sought cutoffs or time points distinguishing uncomplicated recovery from infection (Shen et al., 2019), and sustained postoperative fever and C-reactive protein trajectories have also been evaluated as indicators of infectious complications (Hwang et al., 2020; Mok et al., 2008). However, adjustment for operative characteristics has been inconsistent. Iwata et al. (2016) identified lymphocyte count at POD4 and C-reactive protein (CRP) at POD7 as markers less affected by operative variables. Kraft et al. (2011) reported that BMI > 25 correlates with higher peak CRP, but diagnostic thresholds were not stratified by BMI.

This work highlights the importance of patient and operative context when interpreting early postoperative WBC and NLR. POD3 elevations showed stronger exploratory associations with SWI than immediate POD1 elevations, but those associations weakened after accounting for corresponding POD1 values and remain unvalidated.

### 4.1 Limitations

This study has several limitations. First, it is retrospective and subject to selection bias. Patients with complete paired POD1–POD3 laboratory values had longer operative duration, greater blood loss, longer hospital stay, and more open procedures than patients without complete paired values. The paired trajectory analyses may therefore be most generalizable to patients who remain hospitalized through POD3 testing.

Second, only 18 SWI events occurred, and only 17 had complete POD1–POD3 paired values. This limited covariate adjustment and increased the risk of unstable estimates. Multivariable models were intentionally parsimonious; penalized regression, internal validation, and propensity-score methods should be considered in larger cohorts. Only nine events required return to OR or washout, precluding a stable regression sensitivity analysis restricted to this more definite phenotype.

Third, detailed microbiologic data were not available for all SWI cases. Culture status, causative organisms, polymicrobial infection, antibiotic duration, and standardized infection-depth classification could not be reported consistently. Future studies should distinguish superficial incisional infection from deep or implant-associated postoperative spine infection using standardized criteria.

Fourth, CRP, ESR, procalcitonin, and albumin were not available, limiting comparison with markers commonly used after spine surgery. WBC and NLR are nonspecific and may also reflect urinary tract infection, pulmonary complications, systemic illness, or other postoperative complications that were not fully adjudicated.

Fifth, potential confounders, including ASA class, drain use, wound class, local antibiotic use, instrumentation details, standardized revision status, and immunosuppressive therapy, were not uniformly available. Residual confounding remains possible.

Despite these limitations, the study indicates that routine laboratory values on the first postoperative day are heavily confounded by operative stress and patient factors, while POD3 values may show an exploratory association with SWI. Larger prospective, multicenter studies are needed to validate these findings and determine whether any clinically useful thresholds can be established.

## 5 Conclusions

Among adults undergoing elective short-segment lumbar fusion, POD3 WBC and NLR showed exploratory associations with 90 d SWI after limited adjustment, while POD1 associations were more dependent on patient and operative context. These POD3 associations were attenuated after adjustment for corresponding POD1 values, and neither absolute nor percentage POD1-to-POD3 changes were significantly associated with SWI. These results support further study of context-based interpretation of postoperative CBC markers but do not establish diagnostic thresholds, clinical utility, or a validated prediction model.

## Data Availability

The data underlying this study contain protected health information and are not publicly available. De-identified data may be made available from the corresponding author upon reasonable request and with appropriate institutional approvals.

## Code availability

No custom code was developed for this study. Statistical analyses were performed using IBM SPSS Statistics, version 30.0.0.
